# HLA-DR expression in tumor epithelium is an independent prognostic indicator in esophageal adenocarcinoma patients

**DOI:** 10.1007/s00262-017-1983-1

**Published:** 2017-03-18

**Authors:** Margaret R. Dunne, Adriana J. Michielsen, Katie E. O’Sullivan, Mary Clare Cathcart, Ronan Feighery, Brendan Doyle, Jenny A. Watson, Naoimh J. O’Farrell, Narayanasamy Ravi, Elaine Kay, John V. Reynolds, Elizabeth J. Ryan, Jacintha O’Sullivan

**Affiliations:** 1 Department of Surgery, St. James’s Hospital, Trinity Translational Medicine Institute, Dublin 8, Ireland; 2 Department of Histopathology, Trinity College, St. James’s Hospital, Dublin 8, Ireland; 3 Present Address: Department of Pathology, RCSI Education and Research Centre, Beaumont Hospital, Dublin 9, Ireland; 4 Centre for Colorectal Disease & School of Medicine, St. Vincent’s University Hospital, University College Dublin, Education and Research Centre, Elm Park, Dublin 4, Ireland

**Keywords:** Barrett’s esophagus, Esophageal adenocarcinoma, Inflammation-associated cancer, Prognostic markers, Survival, HLA-DR

## Abstract

Esophageal adenocarcinoma (EAC) is an aggressive cancer with poor prognosis, and incidence is increasing rapidly in the Western world. Measurement of immune markers has been shown to have prognostic significance in a growing number of cancers, but whether this is true for EAC has yet to be evaluated. This study aimed to characterize HLA-DR expression in the esophagus across the inflammation to cancer progression sequence and to assess the prognostic significance of HLA-DR expression in EAC. Tissue microarrays (TMA) were constructed from esophageal tissue taken from patients at different stages in the cancer progression sequence; normal, esophagitis, Barrett’s esophagus (BE), low- and high-grade dysplasia (LGD, HGD) and EAC. HLA-DR expression in tissue epithelium and stroma was assessed by immunohistochemistry. HLA-DR expression increased early in the inflammation to cancer progression sequence; with higher expression detected in esophagitis and BE compared to normal tissue. Patients with low (<50%) HLA-DR expression in the EAC tumor epithelium had significantly worse survival outcomes, compared to those with high expression, in both the tumor core (hazard ratio, HR = 2.178, *p* = 0.024, *n* = 70) and leading edge (HR = 2.86, *p* = 0.013, *n* = 41). Multivariate analysis demonstrated that low HLA-DR expression in leading edge tumor epithelium was an independent predictor of poor survival, associated with a 2.8-fold increase in disease-associated death (*p* = 0.023). This study shows that HLA-DR is an independent prognostic marker in EAC tumor epithelium. This may have implications for patient stratification strategies as well as EAC tumor immunology.

**Electronic supplementary material:**

The online version of this article (doi:10.1007/s00262-017-1983-1) contains supplementary material, which is available to authorized users.

## Introduction

Esophageal adenocarcinoma (EAC) is an aggressive malignancy with poor prognosis and a 5-year survival of <15% [1]. Incidence is increasing in parallel with the growing obesity epidemic [2] and the number of cases in Ireland is predicted to double by 2035 [3]. EAC is an inflammation-driven cancer, as evidenced by the increased incidence observed in patients with the reflux-associated disorder Barrett’s esophagus (BE) [4]. BE is an inflammatory condition, which arises as a result of chronic gastro-esophageal reflux disease (GERD). BE is characterized by intestinal metaplasia, whereby squamous epithelium is replaced by columnar epithelial cells. Patients with BE are enrolled on endoscopic surveillance programs to monitor for early signs of dysplasia and prevent cancer development. Once EAC is established, treatment protocols increasingly utilize multi-modal approaches, where either neoadjuvant chemotherapy alone or combination chemoradiotherapy followed by surgical intervention has become the standard of care. However only 20–30% of patients will experience a complete or partial response to such treatments [5, 6]. The clinical challenge in this area is the identification of factors or events which can predict patient clinical outcomes. In recent years, the infiltrating immune component of tumors has been shown to be an important factor in predicting patient clinical outcomes [7–10]. This has been demonstrated for several cancer types, with a greater amount of infiltrating immune cells being associated with improved patient survival and reduced tumor reoccurrence.

In this study, we examined the role of the antigen-presenting molecule human leukocyte antigen (HLA)-DR in EAC development. HLA-DR is part of the major histocompatibility complex (MHC) class II family of antigen presentation molecules, encoded on human chromosome 6, region 6p21.31. HLA-DR is constitutively expressed on antigen-presenting cells such as dendritic cells, monocytes, B cells and activated T cells, but expression can also be induced in other cell types, including tumor cells, particularly in response to inflammatory conditions [11–13]. HLA-DR expression in tumors has been shown to be positively associated with patient prognosis in some cancers [14, 15], such as colorectal cancer [16–18], gastric cancer [19], squamous cell carcinoma of the larynx [20], but is negatively associated with prognosis in other cancer types, such as glioma [21] and esophageal squamous cell carcinoma [22]. The reason for this discrepancy is unclear, although different groups use different scoring criteria, and tissue-specific differences in HLA-DR expression have also been reported [23].

HLA-DR expression in tumors correlates with the presence of immune cells such as CD16^+^ myeloid cells and T cells [16], and is, therefore, thought to reflect an immunogenic tumor microenvironment, capable of supporting an anti-tumor immune response. Indeed, MHC class II molecules such as HLA-DR are crucial to the development of CD4 T helper cell subsets [24]. However, it is unclear whether HLA-DR upregulation in tumors precedes or is a result of tumor infiltration by immune cells. It is hypothesized that early infiltrating mononuclear cells produce IFN-γ and other inflammatory molecules, which drive HLA-DR upregulation and further amplify the immune response via subsequent activation of effector T cells [12, 25, 26].

In this study, we determined the level of HLA-DR expressed in esophageal tissue compartments at different stages in the inflammation to cancer progression sequence, and assessed the prognostic role of HLA-DR in EAC patients.

## Materials and methods

### Ethical approval

Full ethical approval was granted by the St. James’s, Adelaide and Meath Hospital, Dublin (SJH/AMNCH) ethics committee and the Beaumont Hospital Ethics Board. This study was carried out in accordance with the Declaration of Helsinki ethical principles for medical research involving human subjects.

### Tissue microarray construction

Three sets of TMA were constructed for this study from paraffin-embedded tissue blocks, with pathological expertise from consultant pathologists (Dr. Brendan Doyle, Professor Elaine Kay). TMA set 1 consists of tissue cores taken from a cohort of patients with normal squamous epithelium (*n* = 15), esophagitis (*n* = 32), BE intestinal metaplasia (*n* = 36), LGD (*n* = 16), HGD (*n* = 9), and EAC (*n* = 7). TMA set 2 included tissue cores from EAC patients where tumor, BE lesions and matched normal mucosa were present in resected tissue (*n* = 29) in patients undergoing esophagectomy at Beaumont Hospital, Dublin. TMA set 3 included tissues from 70 EAC patients undergoing surgical resection at St James’s Hospital, using tissue from both tumor core (*n* = 70) and leading edge tissue (*n* = 41). Areas of interest were marked by a pathologist and 0.6 or 1 mm cores were taken from the blocks and TMA were constructed. Several representative cores (mean *n* = 2, range 1–6) were taken from diagnostic biopsies to construct the TMA. 4 μm sections were placed onto Superfrost Plus poly-L-lysine coated glass slides (Thermo Fisher Scientific, IL, USA), and baked overnight at 37 °C in a tissue drying oven (Binder, Tuttlingen, Germany).

### Immunohistochemistry

Antigen retrieval was carried out using Triology^™^ (Cell Marque^™^ Corporation, CA, USA), combining three pre-treatment steps: deparaffinization, rehydration and unmasking. Sections were incubated in diluted Triology^™^ (1:20) in a DYB350 programmable pressure cooker on low pressure for 10 min. Vectastain Elite kits (Vector Labs, CA, USA) were used for all immunohistochemical staining. Tissue sections were incubated in 3% H_2_O_2_ in methanol (Sigma–Aldrich, MO, USA) to quench endogenous peroxidase activity. Sections were washed three times in PBS and blocked with normal serum. Sections were stained using a 1:1000 dilution of HLA-DR (clone TAL 1B5, Abcam, Cambridge, UK) primary antibody. Sections were washed three times and incubated in a 1:400 dilution of biotinylated secondary antibody, then washed again. Sections were incubated with avidin–biotin complex reagent, followed by washing and incubation with diaminobenzidine (DAB) peroxidase solution (Sigma–Aldrich). Sections were rinsed and counterstained in Harris’s haematoxylin (Sigma–Aldrich). Sections were dipped in two separate baths of 100% methanol (Sigma–Aldrich), transferred into two separate baths of xylene (Sigma–Aldrich), before being placed in a bath of xylene overnight. Coverslips were mounted using DPX mountant (BDH Ltd., Dorset, UK). Images were taken using Aperio Scanscope XT digital scanner. Immunohistochemical staining was assessed at 40× magnification in a semi-quantitative manner by two independent observers (Dr. Adriana Michielsen and Dr. Katie O’Sullivan) who were blinded to the pathology and clinical outcome data during scoring. Epithelial and stromal tissue compartments were separately assessed for percentage positive staining. A 50% cut-off value was chosen to define HLA-DR high and low staining levels, based on the median percentage staining observed.

### Statistical analysis

Statistical analyses were performed using GraphPad Prism version 5.0 for Windows (GraphPad software, La Jolla, CA). One-way ANOVA was used to compare HLA-DR expression between groups, and Tukey’s Multiple Comparison Test was used to define significantly differing groups. Cox proportional hazard regression was used to assess the influence of HLA-DR expression on patient survival. A *p* value of <0.05 was considered to be significant. Kaplan–Meier survival curves were drawn for categorical factors and used, along with Schoenfeld residual plots, to verify the proportional hazards assumptions made. Survival was calculated as the time, in months, from the day of diagnosis until death of the patient. The last follow-up date were used as a cut-off time point for survival calculations.

For multivariate analysis, data were analyzed using SPSS version 20.0 (IBM, Armonk, New York, USA). Kaplan–Meier estimates were used to calculate survival curves, differences in survival curves were calculated using log-rank analysis. Cox regression multivariate analysis was used to determine independent predictors of survival; only variables with significance on univariate analysis were input into the multivariate analysis. Statistical significance was defined by *p* value < 0.05.

## Results

### HLA-DR is upregulated early in the inflammation to cancer progression sequence

**Fig. 1:**
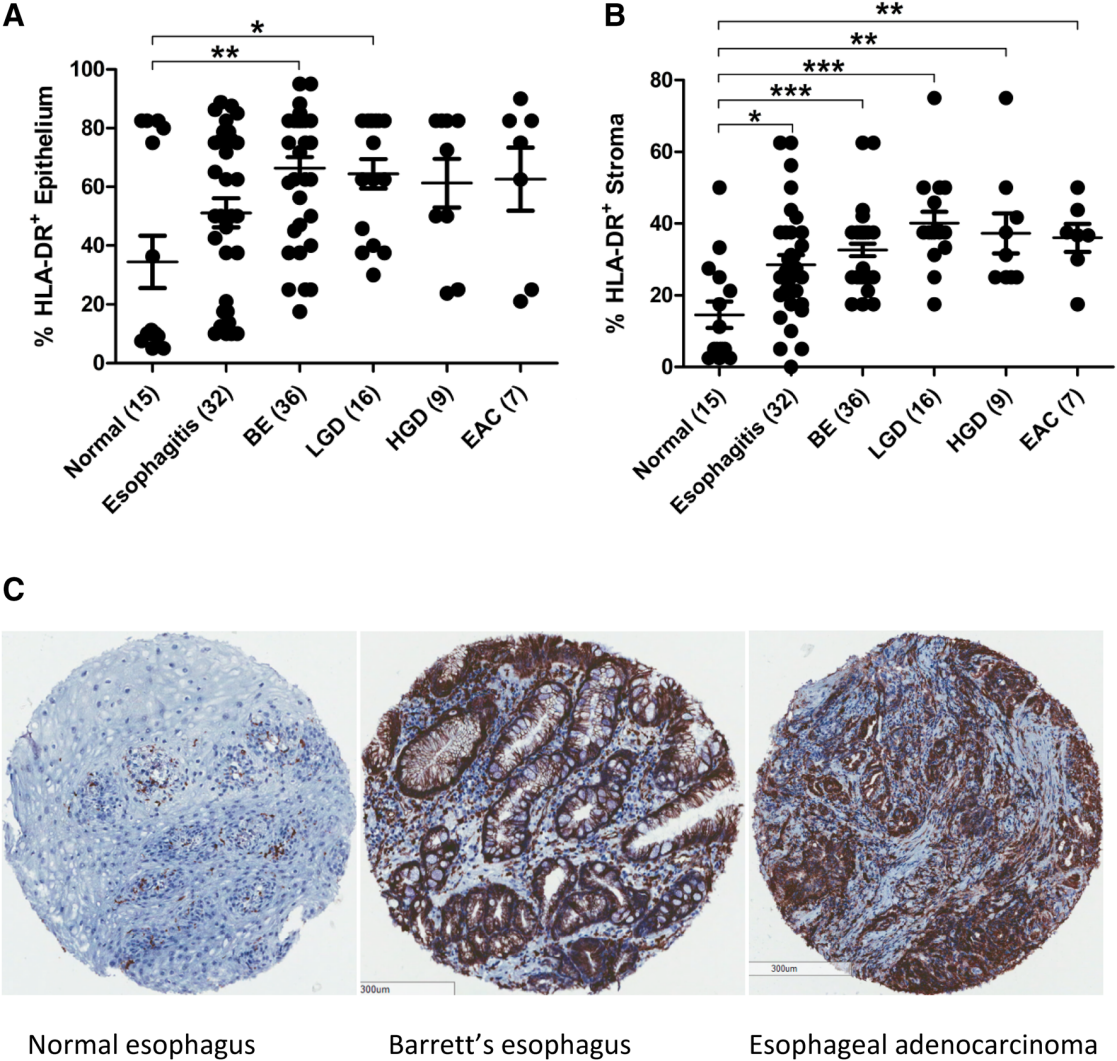
HLA-DR expression is upregulated in esophageal tissue early in the inflammation to cancer progression sequence. HLA-DR is expressed as percentage positive staining in the epithelium (**a**) and stroma (**b**) of normal squamous esophageal tissue from healthy donors (*n* = 15), esophagitis patients (*n* = 32), BE intestinal metaplasia (*n* = 36), LGD (*n* = 16), HGD (*n* = 9) and EAC (*n* = 7) patients. One-way ANOVA was used to compare HLA-DR expression between groups, and Tukey’s Multiple comparison test was used to define significant differences. Representative HLA-DR staining is shown for esophageal tissue TMA at various stages in cancer progression sequence (C). *p < 0.05, **p < 0.01, ***p < 0.001

Figure 1 shows the percentage of HLA-DR positive cells present in the esophageal epithelium and stroma cell compartments in the cancer progression sequence from normal esophagus, esophagitis, BE, LGD, HGD to EAC tumor. HLA-DR expression in the esophageal epithelium is increased in inflammatory states and early dysplasia, but levels remain unchanged upon progression from inflammation to cancer (Fig. 1a). This increase was significant in patients with BE (*p* < 0.01) or LGD (*p* < 0.05), compared to normal squamous epithelium. In esophageal stromal tissue (Fig. 1b), HLA-DR expression is significantly elevated, at every stage of the progression sequence; esophagitis (*p* < 0.05), BE (*p* < 0.001), LGD (*p* < 0.001), HGD (*p* < 0.01) and EAC (*p* < 0.01) compared to normal tissue. In both epithelial and stromal tissue compartments, HLA-DR was upregulated at an early, inflammatory stage and levels remained high during the progression to cancer.

### HLA-DR expression is highest in EAC tumor compared to matched BE or adjacent normal tissues

**Fig. 2:**
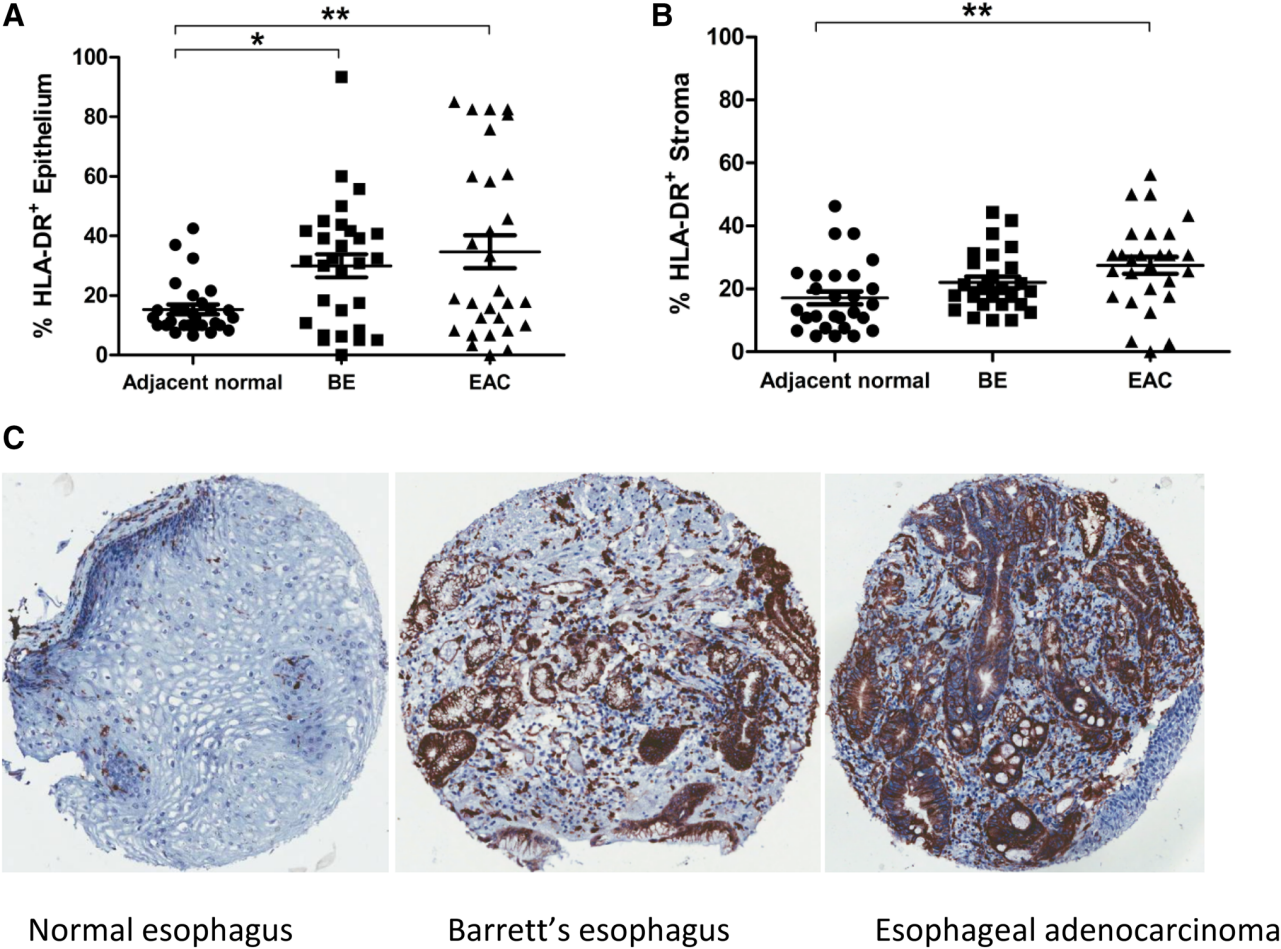
HLA-DR expression is highest in EAC tumor compared to BE or normal tissues from the same donor. HLA-DR percentage positive staining is shown for esophageal tissue epithelium (**a**) and stroma (**b**) at different tissue sites from the same EAC donors (*n* = 29). Representative HLA-DR staining is shown for esophageal tissue TMA at different sites for a single donor (**c**). A repeated measures ANOVA was used to compare HLA-DR expression between groups, and Tukey’s Multiple comparison test was used to define statistically different groups. *p < 0.05, **p < 0.01

For a more in-depth assessment of tissue-specific HLA-DR expression, TMA were also constructed from tissue samples from 29 resected EAC tumor cases with matched BE lesions and normal adjacent tissues, shown in Fig. 2. HLA-DR expression in the esophageal epithelium (Fig. 2a) was evident in adjacent histologically normal tissue (mean percentage positivity 15.3%, range 6.7–42.5%) and was significantly increased in matched BE (mean 30%, range 0–93.3%, *p* < 0.05) and EAC tissues (mean 34.7%, range 0–85%, *p* < 0.01). HLA-DR expression in esophageal tissue stroma (Fig. 2b) followed a similar trend with increasing positivity; although this increase was only significant for EAC tissues (mean 27.5%, range 0–56.3%, *p* < 0.01) compared to normal adjacent tissue (mean 17.1%, range 5–46.3%), but was not significantly altered when compared to BE lesions (mean 22%, range 10–44.3%). No significant differences in HLA-DR expression were observed when BE lesions were compared to EAC tumors, for either the epithelium or stromal compartment.

### EAC patients with a history of BE have higher levels of HLA-DR expression in EAC tumor epithelium

**Fig. 3:**
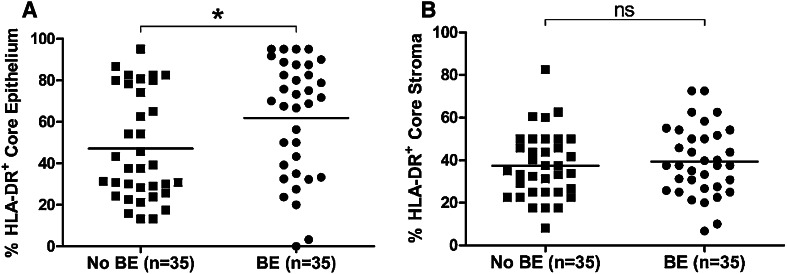
Patients with a history of BE show higher HLA-DR expression in EAC tumor epithelium, when compared to EAC patients with no known history. HLA-DR expression was significantly elevated in tumor epithelium (**a**) of EAC patients with a history of BE (*n* = 35), compared to EAC patients with no known BE history (*n* = 35), but not in stroma (**b**). Differences were assessed by *t* test, *p < 0.05

HLA-DR expression was compared in EAC patients with a history of BE (*n* = 35) versus those with no known history of BE (*n* = 35). HLA-DR expression was significantly elevated (*p* < 0.05) in the EAC tumor epithelium of patients with a history of BE (Fig. 3a). No difference was observed in EAC stroma (Fig. 3b).

### Overall HLA-DR expression pattern is similar between EAC tumor core and leading edge tissues, but higher in tumor epithelium compared to stroma

**Fig. 4:**
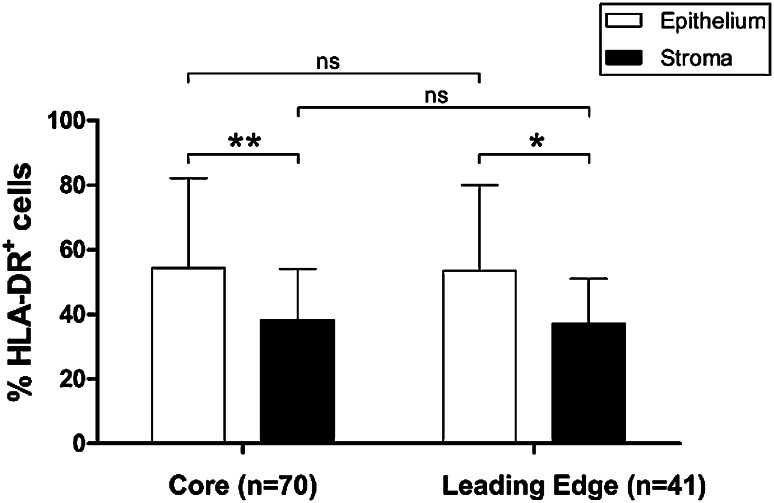
Overall HLA-DR expression pattern is similar between EAC tumor core and leading edge tissues, but is higher in tumor epithelium compared to stroma. HLA-DR percentage positive cells were compared across TMA prepared from EAC tumor core (*n* = 70) or leading edge (*n* = 41) tissue. HLA-DR expression level was also compared as a percentage of positive epithelial cells compared to percentage positive stroma, in each tissue location. Differences in expression levels in different compartments were assessed by Mann–Whitney U test. *p < 0.05, **p < 0.01

Attempts to critically evaluate immune scoring methods used to predict patient prognosis have highlighted the potential for differences in immune scores in different parts of tumor tissue. Galon et al., recommend scoring both the tumor core and invasive margin [9]. We compared HLA-DR expression in the EAC tumor core (*n* = 70) and leading edge (*n* = 41) tissue and found no significant difference in the expression pattern between these different sites, either in tumor epithelium, or stroma (Fig. 4). HLA-DR was more commonly expressed in the EAC tumor epithelium compared to stroma at both the leading edge (*p* = 0.0115) and tumor core (*p* = 0.0012), however.

### Elevated HLA-DR expression in EAC tumor core epithelium strongly correlates with improved patient survival

**Fig. 5:**
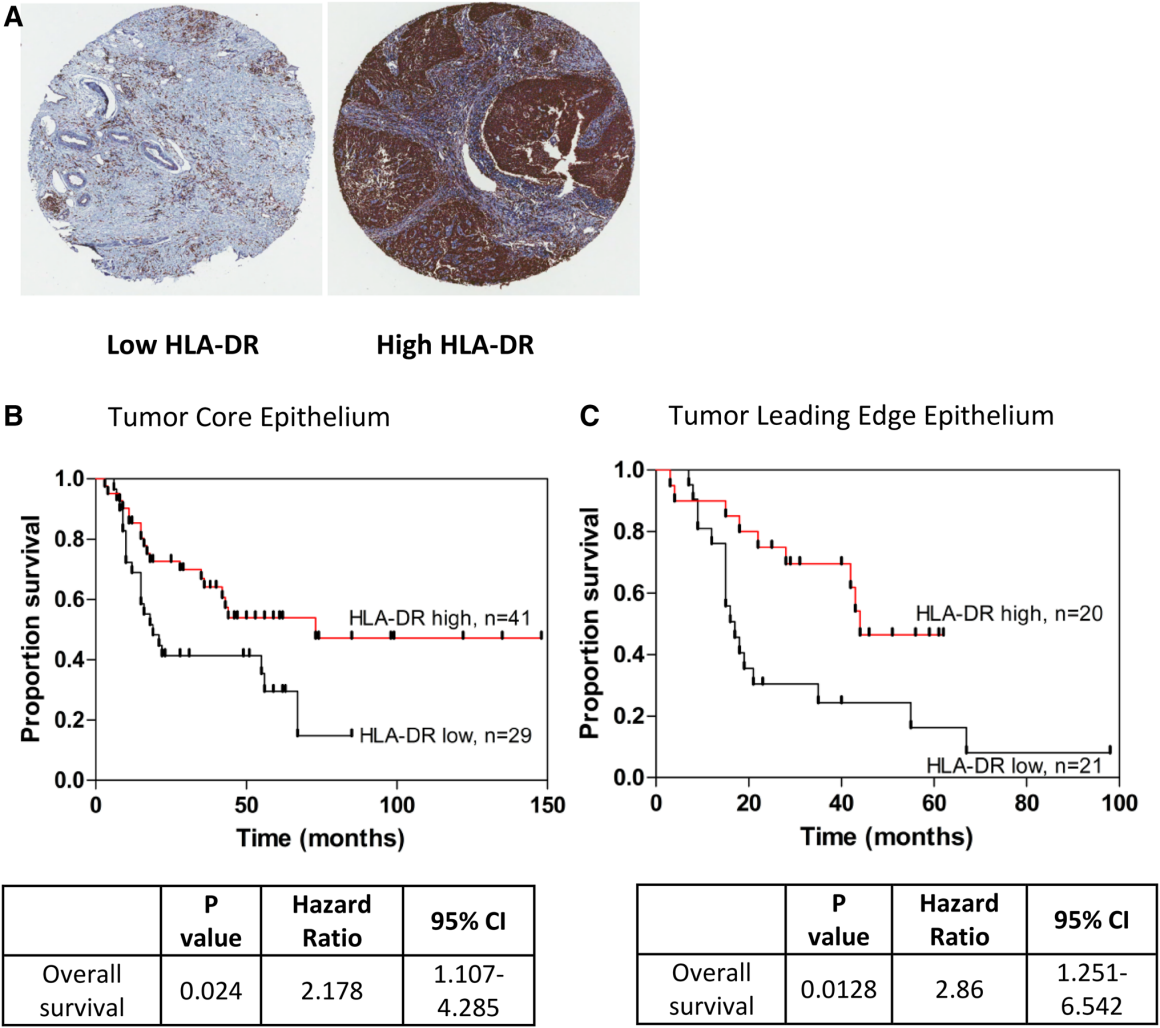
Low HLA-DR expression in EAC tumor epithelium is associated with worse overall survival. Representative HLA-DR staining is shown for TMA constructed from EAC patient tumours (**a**). Patients with low (<50%^+^) HLA-DR expression in the EAC tumor epithelium were more than twice as likely to die than those patients with high (≥50%) expression, as represented by a Kaplan–Meier survival curves, in both the tumor core, *n* = 70 (**b**) and leading edge, *n* = 41 (**c**). Associations were tested using a Log-rank (Mantel-Cox) test

To assess whether HLA-DR expression predicts patient survival the setting of EAC, we assessed a cohort of *n* = 70 EAC patients (see Supplementary Table 1 for patient demographics). Expression was divided into two levels: low HLA-DR expression (defined as 0–49% positive staining) and high HLA-DR expression (defined as 50–100%) and Kaplan–Meier survival analysis was performed for both cohorts. As shown in Fig. 5, patients with low HLA-DR expression in the EAC tumor epithelium had a significantly shorter survival time compared to patients with high expression, in both EAC tumor core (Fig. 5b, *p* = 0.024, HR = 2.18) and leading edge tissue (Fig. 5c, *p* = 0.013, HR = 2.86), upon survival analysis. This survival difference was only evident in the EAC tumor epithelium, however, and was not observed in EAC tumor stroma (Supplementary Fig. 1).

In the tumor leading edge epithelium in particular, low HLA-DR expression was associated with a significantly shorter survival (mean 29.8 months, 95% CI, range 17.5–42.2 months) than patients with high HLA-DR expression (mean 43.4 months, 95% CI, range 34.0–52.8 months), (*p* = 0.013). Furthermore, Cox regression multivariate analysis demonstrated that low HLA-DR expression in leading edge epithelium was the single independent predictor of poor survival, associated with a 2.8-fold increase in disease-associated death (*p* = 0.023), as shown in Table 1.

**Table 1:** Univariate and multivariate analysis examining poor predictors of patient survival

	Univariate analysis *p* value	Multivariate analysis *p* value
Female sex	0.058	–
Normal BMI (18.5–24.9)	0.003	0.077
History of BE	0.102	–
Lymphatic invasion	0.044	0.458
Vascular invasion	0.078	–
Perineural invasion	0.105	–
Differentiation	0.079	–
pT stage	0.224	–
pN positivity	0.001	0.059
Low HLA-DR core stroma, *n* = 70	0.08	–
Low HLA-DR core epithelium, *n* = 70	0.013	0.106
Low HLA-DR leading edge stroma, *n* = 41	0.148	–
Low HLA-DR leading edge epithelium, *n* = 41	0.013	0.023

## Discussion

The presence of functional antigen presentation machinery is of utmost importance in raising an effective immune response against malignancy. The absence or loss of antigen-presenting molecules confers a level of immunological invisibility upon tumor cells, and is associated with tumor progression, metastasis and poor prognosis [27, 28]. Elevation of CD14^+^HLA-DR^−/low^ cells has been described as an indicator of poor prognosis, in terms of patient survival [29], increased metastasis and poor response to chemotherapy [30], whereas elevation of HLA-DR in tumors has been associated with favorable outcomes in several cancer types [17, 19, 20]. We describe here for the first time that elevated HLA-DR expression in tumor epithelium is an independent prognostic marker of patient survival in EAC. This observation agrees with reports in other gastrointestinal cancers such as colorectal cancer [16, 18] and gastric cancer [19] which also show an association between elevated HLA-DR expression in tumors and favorable clinical outcomes.

The association between HLA-DR expression and prolonged survival is not true for all types of cancer however [15], with elevated HLA-DR even reported as a negative prognostic marker for certain cancer types, such as glioma [21]. The reasons for this discrepancy are unclear but could be tissue or cancer type-specific, or alternatively, could potentially be attributable to the types of HLA-DR-expressing cells measured in the tumor microenvironment, i.e., tumor epithelial cells as opposed to tumor stromal or infiltrating immune cells. Interestingly, we only observed a positive prognostic correlation when patient survival was measured against HLA-DR expression in the tumor epithelium, but not in the tumor stroma. This difference in tissue compartments could potentially explain why other studies, which reported a single overall tumor tissue score, failed to show a significant correlation between HLA-DR expression and patient outcome.

In our multivariate analysis, HLA-DR expression in the EAC tumor leading edge was the sole independent prognostic marker of patient survival. Lymph node positivity was significant in univariate (*p* = 0.001), but not multivariate analysis (*p* = 0.059), unlike other studies where lymph node positivity is the single most important prognostic factor [31]. This disparity is most likely due to the relatively small number of patients assessed (*n* = 70). In colorectal cancer, Sconocchia and colleagues report a significant association between low MHC class II expression in tumors and lymph node involvement (*p* < 0.0001) [16]. However, whether the prognostic potential of HLA-DR truly is superior to that of nodal status must be evaluated in a larger patient cohort.

The observation that HLA-DR expression level is only prognostically favorable when measured in EAC tumor epithelial cells suggests an intriguing possibility that tumor epithelial cells may be performing some immunostimulatory function. Indeed, esophageal epithelial cells expressing HLA-DR have been shown to have functional ability and can effectively process and present antigens to T cells [12]. Therefore, it is possible that HLA-DR upregulation by tumor epithelial cells plays a direct functional role in anti-tumor immunity, potentially acting as a compensatory mechanism for tumor-induced inhibition of professional antigen-presenting cells such as dendritic cells, as previously shown by our group in colorectal cancer [32].

Characterization of HLA-DR expression level across the cancer progression sequence shows that HLA-DR upregulation is an early event in inflammation leading to cancer. HLA-DR upregulation in inflammation has been previously reported in ulcerative colitis [11] and esophagitis [12]. This observation is consistent with the hypothesis that HLA-DR upregulation occurs in response to various pro-inflammatory mediators, such as IFN-γ [13, 33]. In the esophagus, T cells have been implicated in the initiation of inflammation, and likely supply these early pro-inflammatory signals [34].

We observed that HLA-DR expression was highest in EAC tumor tissues, when compared to matched inflamed BE lesions or histologically normal adjacent tissue. This was especially true in EAC tumors expressing high (≥50% positive) levels of HLA-DR, all of which showed HLA-DR expression to be highest in the tumor tissue. This finding is similar to observations in colorectal tumors, where both percentage of HLA-DR^+^ cells and staining intensity were highest in tumors compared to colorectal adenomas [16].

Differences have been reported in basal HLA-DR expression level in different areas of the gastrointestinal tract, with small intestinal tissues showing some expression under normal conditions, whereas colorectal tissues typically do not [11, 35]. Reports of HLA-DR expression level in tissues varies widely, partially due to inherent variability in immunohistochemistry methodology and scoring methods. However, we observed clear differences in overall HLA-DR expression level between EAC tumors and colorectal tumor tissue (data not shown), stained using identical protocols. Whereas all but one EAC tumor specimen stained positively for HLA-DR expression, over half of all colorectal tumors scored 0% expression (our unpublished observations). Such variable HLA-DR expression patterns in different tumor types have been noted in other studies, where expression was reported on 12% (*n* = 8/69) of squamous cell carcinomas of the larynx [20], 67% (*n* = 47/70) gastric carcinomas [19], 22% (*n* = 13/60) non small cell lung cancer cells [36], and 45% (*n* = 49/108) esophageal squamous cell carcinomas [22].

Aside from survival, HLA-DR expression level in the tumor core (*n* = 70) was not significantly associated with changes in other clinical parameters tested (Supplementary Fig. 2), such as pathological tumor stage (T1–T4) (Supplementary Fig. 2a and b), even when high (T3/T4) versus low (T1-T2) stages were grouped for comparison (data not shown). Similarly, HLA-DR expression levels were not significantly altered when EAC patients were segregated by tumor differentiation status (Supplementary Fig. 2 c and d), lymph node positivity (Supplementary Fig. 2e and f), perineural involvement (data not shown) or overall T stage (data not shown). Similar results were observed in EAC tumor leading edge tissue (*n* = 41, data not shown). In terms of future work, it will be of great interest to determine whether HLA-DR expression is associated with EAC patient response to neoadjuvant therapy. Multi-modal neoadjuvant therapy is rapidly becoming the standard treatment option for EAC patients, yet only a minority of patients will respond, meaning the majority of patients undergo this treatment and its associated side effects without benefit. Investigating the immunological basis of this response to treatment will be critical in advancing the understanding and potential future manipulation of immune responses to eliminate established tumors.

In summary, we show that high HLA-DR expression in the EAC tumor epithelium is an independent favorable prognostic indicator of prolonged patient survival, and screening for HLA-DR may be a useful addition to the growing number of prognostic immune-based tests.

### Electronic supplementary material

Below is the link to the electronic supplementary material.

Supplementary material 1 (PDF 258 KB)

## Abbreviations

BE: Barrett’s esophagus
CI: Confidence interval
DAB: Diaminobenzidine
EAC: Esophageal adenocarcinoma
GERD: Gastro-esophageal reflux disease
HGD: High-grade dysplasia
HR: Hazard ratio
LGD: Low-grade dysplasia
pN: Pathological lymph node stage
pT: Pathological tumour stage
TMA: Tissue microarrays
