# Supplementary material for: HLA-DR expression in tumor epithelium is an independent prognostic indicator in esophageal adenocarcinoma patients
Source: Cancer Immunol Immunother. 2017 Mar 18;66(7):841–50. doi: 10.1007/s00262-017-1983-1 (PMC5489642; doi:10.1007/s00262-017-1983-1)
Supplement: Supplementary file 1 — Supplementary material 1 (PDF 258 KB) [file 262_2017_1983_MOESM1_ESM.pdf]

Supplementary table 1 Demographic data for survival analysis	
Male/Female	59/11
Median age at diagnosis (range)	68 (38-84)
Clinical stage of disease	
Stage 0	1
Stage 1	10
Stage 2	31
Stage 3	19
Stage 4	1
Unknown	8
Pathological stage of disease	
Stage 1	12
Stage 2	20
Stage 3	33
Stage 4	4
Unknown	1
Median overall survival in months (range)	28.5 (3-148)
Known history of BE	35

A **Tumor Core Stroma**

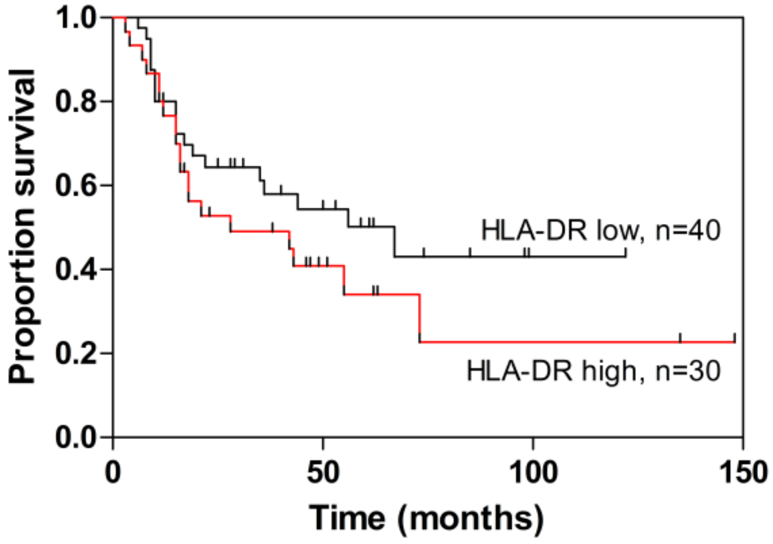

B **Tumor Leading Edge Stroma**

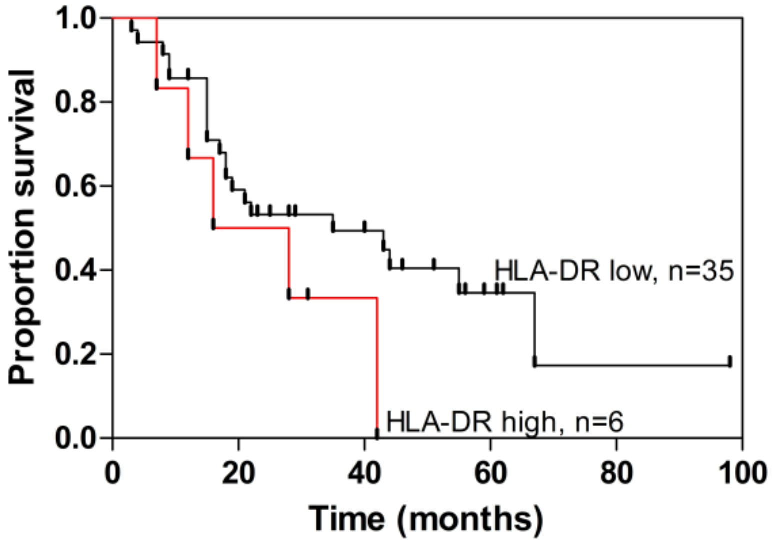

Supplementary figure 1 HLA-DR expression level does not predict survival outcome when measured in the EAC tumor stroma

No significant difference was observed in the risk of mortality between patients with low (<50%) HLA-DR expression compared to those with high (≥50%) expression in the EAC tumor stroma, either in the tumour core (A) or leading edge (B). Associations were tested using a Mantel-Cox log-rank test.

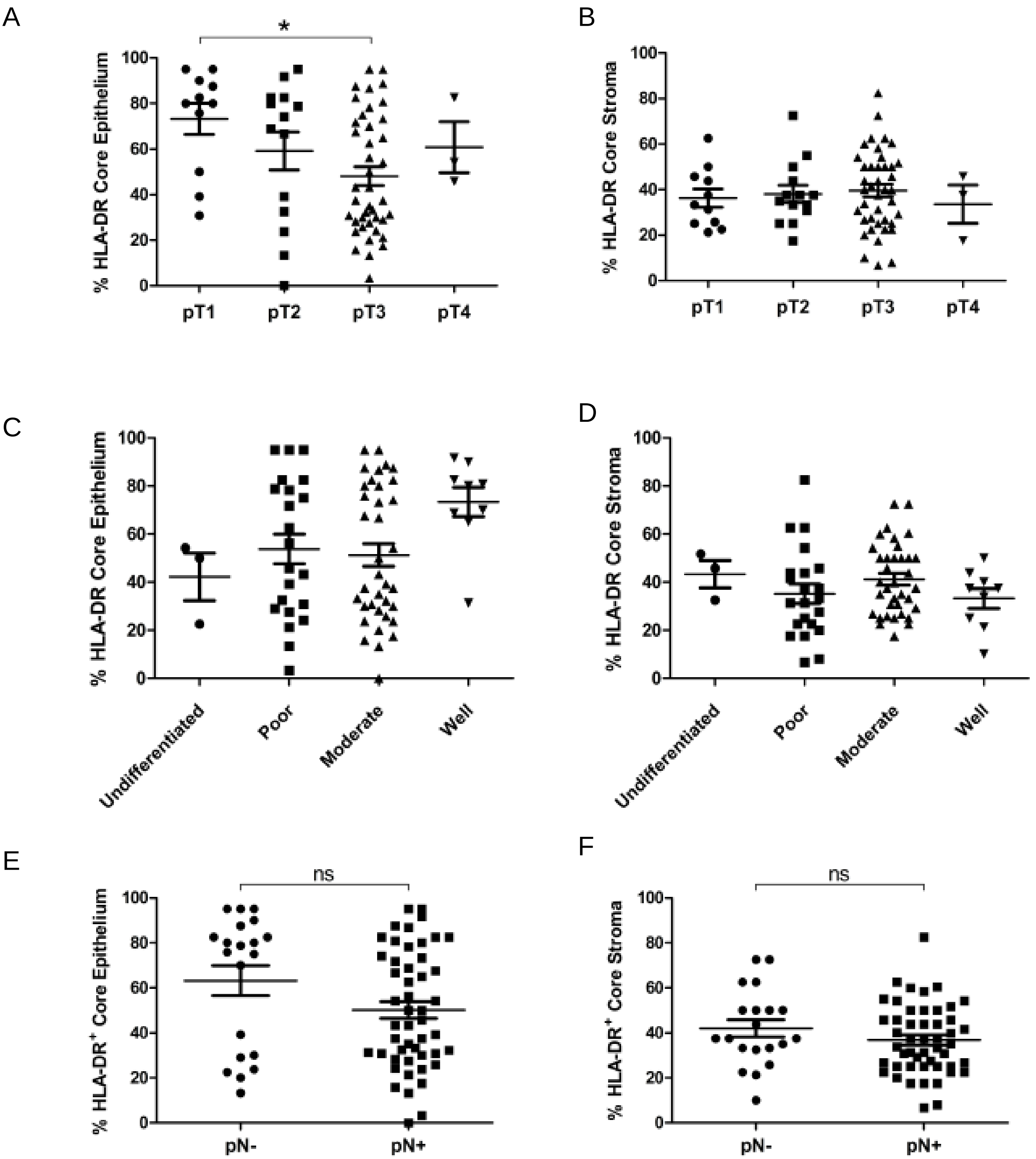

Supplementary figure 2 Pathological correlates with HLA-DR

HLA-DR levels overall did not change significantly with changes in pathological T stage (A, B), tumor differentiation status (C, D) or lymph node involvement (E, F), in either tumor epithelium or stroma, respectively. Similar results were seen for EAC leading edge tissue. pT = pathological T stage, pN = pathological lymph node positivity. Associations were tested by *t* test or Kruskall-Wallace and Dunn's Multiple Comparisons tests, as appropriate, **p*<0.05.
